# Evaluating Language Models for Biomedical Fact-Checking: A Benchmark Dataset for Cancer Variant Interpretation Verification

**DOI:** 10.1101/2025.09.10.675443

**Published:** 2025-09-15

**Authors:** Caralyn Reisle, Cameron J. Grisdale, Kilannin Krysiak, Arpad M. Danos, Mariam Khanfar, Erin Pleasance, Jason Saliba, Melika Hanos, Nilan V. Patel, Asmita Jain, Joshua F. McMichael, Ajay C. Venigalla, Malachi Griffith, Obi L. Griffith, Steven J. M. Jones

**Affiliations:** 1. Canada’s Michael Smith Genome Sciences Centre; 2. Bioinformatics Graduate Program, Faculty of Science, University of British Columbia, Vancouver, BC, Canada; 3. Department of Pathology and Immunology, Washington University in St Louis School of Medicine, St. Louis, MO, USA; 4. Siteman Cancer Center, Washington University in St Louis School of Medicine, St. Louis, MO, USA; 5. McDonnell Genome Institute, Washington University in St Louis School of Medicine, St. Louis, MO, USA; 6. Department of Medicine, Washington University in St Louis School of Medicine, St. Louis, MO, USA; 7. Department of Genetics, Washington University in St Louis School of Medicine, St. Louis, MO, USA; 8. Department of Medical Genetics, University of British Columbia, Vancouver, BC, Canada

## Abstract

Accurate interpretation of genomic variants is critical for precision oncology but remains slow and dependent on specialized expertise. Public knowledgebases such as the Clinical Interpretation of Variants in Cancer (CIViC) help by curating literature-backed variant interpretations in a structured form, yet verification and review have become major bottlenecks. To address this, we developed CIViC-Fact, a benchmark dataset and pipeline for testing automated systems that verify the accuracy of cancer variant claims. CIViC-Fact links structured claims to sentence-level supporting or refuting evidence from full-text articles, and includes expert annotations and explanations.

We evaluated multiple language models. Proprietary models performed well without training, but a smaller open-source model, fine-tuned on CIViC-Fact, achieved the highest accuracy (89%). Applying our fact-checking pipeline to real CIViC entries showed that reviewing less than 20% of content, focusing on flagged entries, would be sufficient to catch over half of all errors. This AI-assisted triage greatly accelerates the review process without replacing or reducing expert insight, ensuring that existing careful oversight remains in place while curators can work more efficiently. CIViC-Fact provides a realistic, high-consequence framework for biomedical fact-checking and a path toward more rigorous and efficient knowledgebase curation.

## Introduction

Tailoring treatment to an individual's genetic profile through precision oncology has played an increasing role in cancer treatment over the last decade. Research has shown value in approaching cancer on a case-by-case, personalized basis^1–5^. However, its translation into standard of care remains constrained. The major obstacle lies in variant interpretation. Analysis requires deep expertise in cancer biology, genomics, and bioinformatics^6^ making it slow, resource-intensive, and difficult to scale

To mitigate repetitive case-by-case literature review, knowledgebases such as OncoKB^7^, CIViC^8,9^, and others^10–17^ curate structured, literature-backed variant annotations, enabling programmatic access to actionable insights^18^. Among these, CIViC (Clinical Interpretation of Variants in Cancer) is unique in being openly accessible, crowdsourced, and allowing anyone to contribute or edit entries enabling rapid growth through community contributions. Yet, ensuring the accuracy and auditability of data that may inform treatment decisions remains paramount^19^. Content must be reviewed by a small team of expert editors, and this verification stage has become the rate-limiting step, creating a new major bottleneck in knowledgebase curation.

Natural language processing (NLP), the AI-driven analysis of human language, has shown promise in precision oncology applications, including variant classification^20^, relation extraction^21–23^, and others^24–26^. Yet, no solution addresses the efficiency of reviewing submitted content, a high-stakes task akin to scientific fact-checking. Large language models (LLMs) show promise in generalizing to biomedical domains^27^. However, their unreliability (hallucinations, factual errors)^28^, and lack of transparency makes them unsuitable for deployment without rigorous evaluation.

Here, benchmarks play a crucial role. Human expert curated benchmarks (e.g. BLURB^29^, PubMedQA^30^) are essential in enabling fair comparisons and tracking of real progress. Most existing fact-checking benchmarks are based on general^31,32^, or political knowledge^33^ and are unsuitable for fact-checking in a scientific context. Scientific publications are particularly challenging, as they rely on technical language, jargon, and domain-specific expertise that make it difficult for NLP models to verify claims. Existing scientific fact-checking datasets are further limited by relying primarily on abstracts, overlooking claims that can only be verified using full-text evidence^30,34^. This limitation is critical: in the CIViC knowledgebase, 61% of clinically relevant claims depend on experimental results or tables not present in abstracts. Yet working with the full-text introduces significant challenges to language models, which often struggle with longer inputs^35^. To add to this complexity, precision oncology utilizes numerous domain-specific synonyms, aliases, and nomenclature that evolve with time. No benchmark currently exists for evaluating fact-checking in biomedical knowledgebases, leaving a critical gap at the intersection of AI and precision oncology.

To address this gap, we present CIViC-Fact, an expert-curated benchmark dataset built to advance automated fact-checking and claim verification in the biomedical domain. CIViC-Fact brings several innovations that make it a powerful resource for evaluating biomedical language models, especially in precision oncology. Unlike most existing science-focused NLP datasets^30,34^, which rely mainly on abstracts, CIViC-Fact includes evidence drawn from the full text of biomedical publications and incorporates both structured and unstructured evidence, such as tabular data and free text, reflecting the heterogeneous formats used in biomedical literature. Claims are written in a structured (tabular) format aligned with the CIViC data model, allowing for precise and interpretable fact-checking. Given the complexity of variant interpretation, annotations in CIViC-Fact were generated by 13 expert annotators from multiple institutions achieving a high level of agreement (77.9%). This high agreement shows that, with sufficient context, experts can reliably judge claim veracity, setting a clear benchmark for models. By incorporating variant interpretations, full-text evidence, and expert-validated gold labels, CIViC-Fact offers a challenging and realistic benchmark for the development and evaluation of trustworthy biomedical language models.

## Methods

### Collection of Sentence Level Evidence Provenance

**Figure 1.**
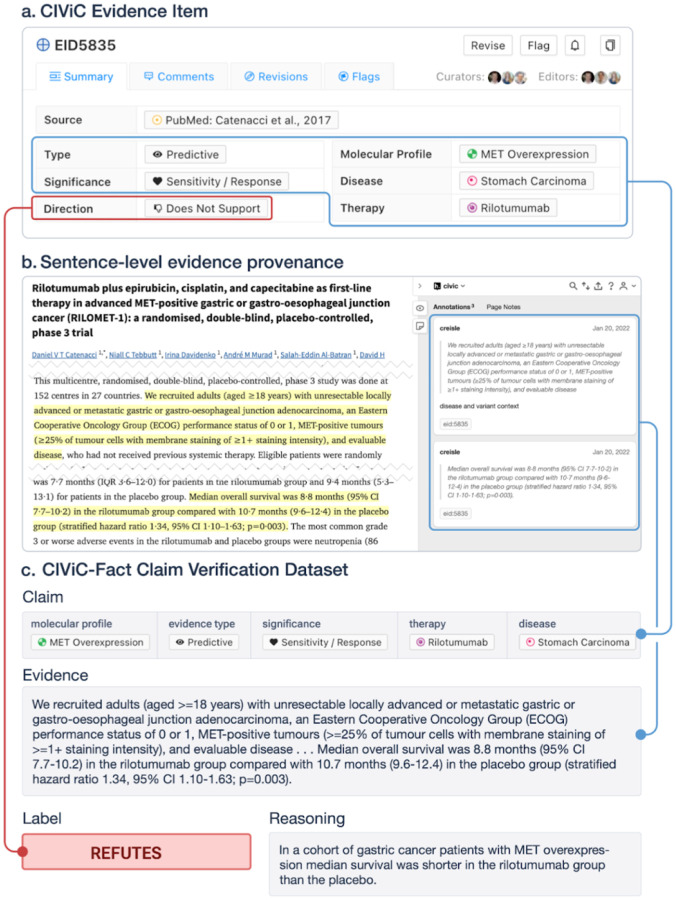
An example entry in the CIViC-Fact dataset. A) The claim is composed from the structured elements of a CIViC entry: molecular profile (gene and variant), disease, therapy, type, significance, and phenotypes. B) Evidence was selected from PubMed Central (PMC5898242) by a curator using the web-tool hypothes.is. C) Stance label is derived from the evidence direction in CIViC resulting in an entry in CIViC-Fact with claim, evidence, and stance label components.

CIViC collects variant interpretations from literature stored as *Evidence Items*. Each *Evidence Item* in CIViC links a scientific article to a variant interpretation with predictive (therapeutic), diagnostic, prognostic, predisposing, oncogenic or functional significance. CIViC *Evidence Items* include both positive and negative findings depending on the *Evidence Direction* (supports or does not support respectively). CIViC-Fact was constructed by aligning CIViC’s curated *Evidence Items* (Fig 1a), hereafter referred to as claims, with the full-text scientific articles from which they were originally derived. CIViC-Fact augments claims in the CIViC knowledgebase with sentence-level evidence provenance, linking each claim to the specific sentence spans in the article that support or contradict it using the tool hypothes.is(Fig 1b). After data was collected by annotators, it was processed and formatted. Tagged annotations were downloaded via the hypothes.is API along with CIViC entries via the CIViC API. Articles were then matched to their full-text XML version from pubmed/PMC based on the article referenced by the CIViC entry. XML articles were converted to the standard Bioc format^36^ using the parsing package developed as part of CIViCmine^26^ (https://github.com/jakelever/biotext). The Bioc format was not used directly from PMC as it excluded elements of interest. Text from the hypothes.is annotations were matched to text in the Bioc articles (removing whitespace errors and other formatting issues from the data collected by hypothes.is).

Claim verification is a crucial step in fact-checking where the verifier is given both the claim and relevant evidence (extracted sentence spans) and asked to label the stance of the evidence toward the claim. Following the design of other stance classification and fact-checking datasets^32,34,37–39^ we classify claim/evidence pairs with 3 labels: SUPPORTS, REFUTES, or not enough information (NEI) (Supplementary Table 1). We use the NEI label when the evidence is insufficient to determine the stance of the evidence toward the claim. The gold label for each claim/evidence pair is based on the *Evidence Direction* curated in CIViC (Fig 1c), unless it is overridden by an explicit label specified by the annotator during tagging (e.g. NEI). In order to support claim verification, we augment the dataset with negative examples generated both through modification of gold claims^40^ as well as fully-generated examples^39^.

### Simulating Curation Errors with Generated Negatives

There are a number of different ways errors can happen in CIViC and other cancer knowledgebases beyond simple curator mistakes. There is a level of nuance where a curator may enter data that is not completely incorrect but would be better represented by a different ontology term or is missing a concomitant variant, etc. In order to train a discriminator model that is sensitive to such changes we augment the CIViC-Fact dataset in several ways (Supplementary Fig 1).

In order to generate claim/evidence pairs which represent common curator errors in CIViC curation, we generate NEI examples by modifying the disease term of gold-pairs. CIViC uses the disease ontology^41^ to curate disease terms. Since the disease ontology includes hierarchical disease relationship information, we are able to automatically modify the existing disease term in a claim with a more or less specific term which would require revision if entered in CIViC.

Claims may be supported by different parts of the paper and evidence supporting a claim may be re-written or summarized in several different ways that are all valid. Curators adding content to CIViC curate a written statement along with the structured elements summarizing the evidence in the paper related to the claim. Naturally these statements do not always mention all elements of the claim as they are partly redundant with the structured elements. We include the structured claim paired with the written statement as additional claim verification pairs. However, we first process these written statements with PubTator^42^ and matching via regular expressions to determine if the written statement is sufficient evidence for the structured claim and we include it as a negative NEI example when it is not.

While CIVIC does curate negative findings, these are less common than the positive findings both in CIViC itself and more generally in scientific publications. To address this imbalance, we simulated additional REFUTES examples by modifying the significance term in the claim with its opposing term where such a mapping was possible (Supplementary Table 2).

It is also possible for human curators to enter content incorrectly, misunderstand the CIViC data model, or even link to the wrong source document. Therefore we generated additional negatives to represent such cases by creating plausible claims based on the variants, diseases, and therapies found in the paper (via PubTator^42^) randomly paired with *Evidence Type* and *Significance*. The generated claims were then paired with sentences in the paper using the biomedical passage retrieval model, Specter^43^. The amount of evidence was chosen to match the distribution of selections curated in the gold-evidence.

Claim/Evidence Pairs were partitioned into training (60%), development (20%) and test (20%). Pairs were partitioned by stratified random sampling to ensure representation of all document and claim types. Stratification included document journal and *claim type/significance*. A document was limited to a single partition so claims from the same document would be in the same partition to avoid possible information leakage due to shared evidence selections across claims. Similarly, pair IDs were kept across dataset builds, and manuscripts were kept within a partition to avoid information leakage.

### Expert Human Review of the CIViC-Fact Claim Verification Dataset

As the CIViC-Fact claim verification dataset now contains both gold-labelled and generated pairs we conducted several rounds (Supplementary Table 3) of annotator review to validate the stance label amongst expert annotators as well as assess the quality of the generated content. Expert annotators were given the claim and evidence and asked to give the stance of the evidence toward the claim as SUPPORTS, REFUTES, or NEI. We conducted this analysis using the open source text annotation tool doccano^44^. Pairs for review were selected by stratified random sampling in order to ensure pairs from each class label and partition (where applicable) were included in the chosen subset. We also aimed to maximize diversity in the source publication of the evidence, avoiding additional claims from the same publication (CIVIC may include multiple entries from the same publication if, for example, there are multiple mutations evaluated in the publication). We measured Inter-Annotator Agreement (IAA), the extent to which different annotators agree when labeling the same data, with Fleiss’ kappa score since we have more than 2 annotators. A subset of claim/evidence pairs were annotated with a written explanation of the stance label.

### Benchmarking Passage Retrieval

**Figure 2.**
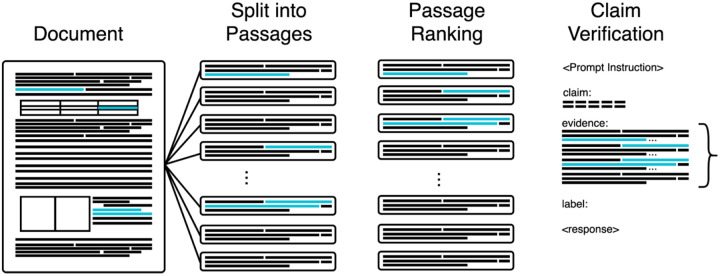
Retrieval of relevant document passages as evidence for claim verification. Relevant selections are shown in blue. A document is split into passages which may or may not overlap content relevant to the claim in question. A passage ranking model is used to order the chunks according to their relevance to the claim. An ideal ranking will put all passages overlapping relevant content at the top of the ranking. The claim and the top-k passages are concatenated as input for claim verification.

To fact-check new CIViC claims efficiently, the pipeline needs an automated step for selecting relevant passages from the source paper, rather than relying on manual curation. Passage retrieval is the process of identifying text segments (passages) that support a claim within a document or a set of documents. Since CIViC already links each claim to a specific paper, our goal is to extract only the passages from that paper that are directly relevant to the claim. Passage embedding models are commonly used for such a task^43,45–47^.These models take as input a query (the claim) and a candidate passage (the potential evidence) and encode them into numerical representations, allowing the model to assess how relevant each passage is to the claim. The fine-grained traceability built into the CIViC-Fact dataset enables systematic evaluation of embedding models, often used for passage retrieval in retrieval-augmented generation (RAG^48^) pipelines, for their effectiveness in selecting appropriate supporting passages from source articles. Each model is given a claim and a list of passages from the evidence and asked to rank the passages in order of their relevance to the claim. The top-ranking passages are then used as evidence input to the claim verification model (Fig 2). We evaluated several popular general and biomedical-specific embedding models (Specter2^49^; MedCPT^45^; and BGE reranker^46^) in addition to the BM25^50^ standard baseline.

From the CIViC-Fact dataset, only provable claims (excludes NEI or claims with conflicting evidence) with access to the full text were used in evaluating passage retrieval. Hereafter, we will refer to this as the CIViC-Fact retrieval subset (n_train_=3456, n_dev_=1194, n_test_=1120). We generated passages by preprocessing manuscripts and splitting them into 1000 character passages with a 100 character overlap. Claim/passage pairs were then ranked and the top-k passages were selected as evidence. The resulting prompt context was provided to the LLM along with a prompt instruction for response generation (Fig 2). Although it is more efficient, bi-encoding (encode the claim and passages separately), degraded performance drastically (data not shown) and it is not necessary as we are only encoding the passages of a single paper at a time, therefore we chose to evaluate cross-encoding models specifically. We evaluated the performance using mean average precision (mAP^51^). mAP measures both the relevance of recommended items and the quality of their ranking.

### Benchmarking Claim Verification Models

**Figure 3.**
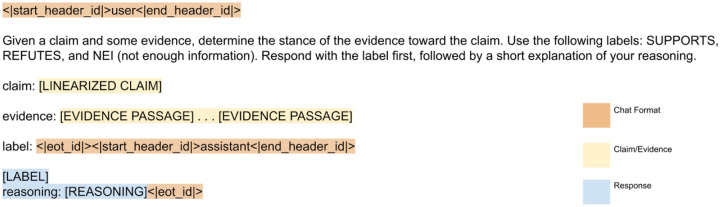
LLM Prompting Format. The prompt begins with an instruction, followed by the claim and evidence pair. Elements specific to a claim/evidence pair are given in with placeholders in uppercase surrounded by square brackets and highlighted in blue or yellow. The expected response from the LLM is highlighted in blue. Chat format elements specific to a particular model (in this case Llama3) are highlighted in orange.

In order to be able to include the structured claim as text input to a language model it must first be preprocessed. The core elements (molecular profile [gene and variant], disease, therapy, significance, and phenotypes) from the CIViC entry were linearized to create the claim. Multiple methods of linearization^52^ were tested but did not significantly affect results (data not shown) and required table parsing which can introduce many errors on the wide variety of table layouts seen in scientific articles. Therefore, we represent tables in a tab-delimited format that is easily human-readable and does not require significant table preprocessing. Several prompt instructions were tested for each model during the initial experiments (data not shown). The most notable difference occurred when we specified that the output label should appear before any other output. Without this instruction, some models failed to include the label within the response due to token limits. For evidence construction, the matched text from the hypothes.is annotations were concatenated in order of appearance, with ellipsis delimiters. The exact prompt instructions and formatting are shown in Figure 3.

We benchmarked a range of language models on CIViC-Fact to assess their ability to verify claims in the CIViC-Fact dataset. Models were given the claim and evidence as input and asked to generate a label and explanation. LLMs that are trained to follow instructions for a variety of tasks use a specific format to denote turns in conversations called chat formats. Chat formats were applied using the transformers^53^ package. Proprietary models were queried using the openai python package. Our experiments included:

- Open LLMs including LLaMA-3.1^54^ (8B, 70B), Mixtral^55^ (8×22B) and BioMistral^56^ (7B), evaluated in zero-shot or few-shot (0–6 examples)
- Fine-tuned Open LLM: LLaMA-3.1 (8B)
- Proprietary LLMs: Gemini-2.5^57^, and GPT-4.1^58^ evaluated in zero-shot settings
- Fine-tuned encoder-based language models trained for stance classification only: BERT^59^ (~340M), BioMedBERT^29^ (~110M), Multilingual-E5^60^ (~600M)

Local fine-tuning (FT) and evaluation on the CIViC-Fact dataset was completed on up to 8 A6000 GPUs or 2 A100 GPUs depending on availability. Due to resource constraints, each model was given a limit of 100 new tokens for each generation and locally run models were 4bit quantized. Proprietary models were also limited to 100 new tokens. PEFT^61^ and qLORA^62^ were used in fine-tuning Llama-3.1 8B. The model was trained for completion only^53^ up to 3 epochs with a learning rate of 0.0002 and we used early stopping to avoid overfitting (Supplementary Fig 2). We have made all training code and related configurations available here (https://github.com/creisle/civicfact/tree/master/experiments). Additionally the fine-tuned versions of models used in this paper are published to Hugging Face hub (https://huggingface.co/docs/hub).

### Evaluating Curation Errors in CIViC

The fact-checking pipeline with the BGE passage retrieval model and Llama FT claim verification model was applied to all claims in CIViC where the full-text is available in PMC. In order to account for non-deterministic results from LLMs, we prompted the model 3 times (3 possible labels) for each claim/evidence pair. If the model predicted the expected label (the label matching the CIViC *Evidence Direction*) all 3 times, then we determine that the fact-checking was able to verify that claim. However, if the model produces an unexpected label, we consider the claim to be flagged by the LLM and we expect there to be potential errors in the content. We manually reviewed a randomly selected subset of both flagged and unflagged content to determine the rate at which flagging resulted in actual errors in CIViC being detected. We also manually reviewed all flagged content.

### Expert Human Review of LLM Explanations

In order to evaluate the explanatory capabilities of LLMs for this task, expert human annotators manually reviewed LLM-generated responses. Annotators were given a claim/evidence pair and the responses from 4 different LLM systems (Llama 8B FT, Llama 70B, GPT-4.1, and Gemini-2.5). The annotators were asked to both generate a gold label and an explanation for the pair (to be used in future training and automated evaluations) as well as evaluate the responses on the following categories (Table 1). Any cases where the newly generated consensus gold label did not match the expected label were flagged and re-examined to ensure high quality gold explanations and labels.

To resolve disagreements from the initial round of tagging, and to capture additional tags such as “*incomplete”* and “*misleading, or overstated”*, each response, along with its initial tags, was re-examined by an additional annotator or collaboratively discussed by the group. We report results for each LLM from this additional analysis. We categorized responses into four groups based on their tagging results: (1) Major Issues, if any major issues were identified; (2) Helpful (Minor Issues), if the response was considered helpful and only minor issues were tagged; (3) Helpful, if the response was helpful with no issues identified; and (4) Unhelpful for all remaining responses.

## Results

### The CIViC-Fact Dataset

Expert annotators manually curated 1,434 claim/evidence pairs covering 1,119 CIViC claims from 554 different publications. CIViC claims paired with their written descriptions were included as further examples. The dataset was augmented with generated negatives to mimic typical CIViC curation errors (Table 2).

To benchmark reliability of human gold labels, we calculated the Inter-Annotator Agreement (IAA) on claim verification judgments. We compute the level of agreement among annotators using both Fleiss’ kappa^63^ (FK) and percent agreement (PA). Across multiple rounds of manual review (Supplementary Table 3) the agreement with respect to evidence stance label was substantial^64^ (FK^63^: 0.65, PA: 77.9%, p-value: 0.0). The high agreement levels suggest that despite the complexity of biomedical literature, human experts can often reach consensus on claim veracity given appropriate context, providing a valuable benchmark for model performance. In nearly all cases (88.1%, Supplementary Fig 3a), disagreements between annotators were between NEI and one of the other labels. For example, annotators disagreed about when details of the claim could be reasonably inferred from the evidence or required more explicit mention in the source text (Supplementary Fig 3b).

### Passage Retrieval Models Perform Well without Fine-Tuning

To fact-check CIViC claims at scale, we incorporated automated passage retrieval to identify the most relevant text segments from the source paper, rather than relying on manual curation. Using the linked source paper for each claim, embedding models ranked candidate passages by relevance, with the top results provided as evidence for claim verification (Fig 2). We compared general and biomedical-specific models (Specter2^43^, MedCPT^45^, BGE reranker^46^) against the BM25^50^ baseline.

**Figure 4.**
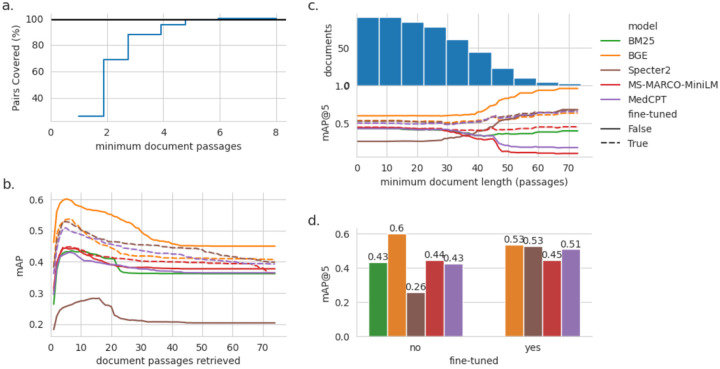
a. Number of passages overlapping manually annotated evidence passages for each example in the retrieval subset of the CIViC-Fact dataset. The 98th percentile is given with a horizontal black line. b. Impact of number of passages retrieved (k-value) on mean average precision. c. Retrieval accuracy (k=5) as measured by mean average precision (mAP) with increasing minimum document length. The reverse cumulative number of documents at each level of minimum length requirement is given by the histogram above the line plot. d. mAP of passage retrieval models at k=5 for all documents.

We measured the performance of passage retrieval models using the mean average precision (mAP) which scores rankings of passages based on the position of relevant documents. A score of 1 indicates that all of the top-k passages retrieved are relevant. The BGE reranker^46^ outperformed the other passage retrieval models even with fine-tuning (see methods), particularly in the longer documents (Fig 4c). Therefore we chose the BGE reranker model for downstream analysis.

While mean average precision (mAP) provides a measure of how relevant the retrieved passages are, it does not indicate whether those passages are sufficient to verify a claim. Scientific articles often contain redundant statements, and a claim may be supported by many possible combinations of disjointed passages. Because our dataset is not exhaustive, we can only approximate retrieval success. Some passages retrieved by the model but not annotated in the dataset may in fact be relevant, false negatives that could serve as valid substitutes for the annotated passages. Therefore, in order to better determine an accurate estimate of the true retrieval accuracy, we refined this estimate through manual review (Supplementary Table 3). For LLM-evaluation and manual review, we chose to limit the retrieval to the top 5 passages as this covers the required selections for 98% of the dataset (Fig 4a). Based on manual review, the BGE model achieved a 93.3% (70/75) rate of successful retrieval.

### Fine-tuning is Required for Accurate Claim Verification

Claim verification is defined as a model’s ability to correctly label the stance of evidence toward a claim when given both the claim and evidence as input. In order to get a measure of language models reasoning performance when the expected evidence was provided, we evaluated language models on the CIViC-Fact dataset with the gold-retrieved evidence collected from hypothes.is or simulated error examples (see methods).

**Figure 5.**
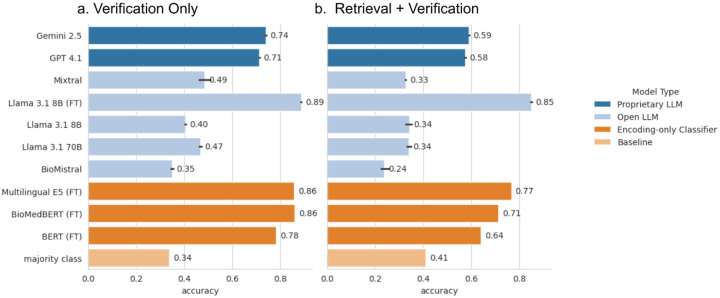
a. Performance of various LLM and BERT-style models for claim verification (stance classification) labelling on the gold-retrieved evidence. The majority class is given as a baseline metric. As LLM outputs are not fixed, each LLM was evaluated on the dataset multiple times to get an average accuracy. Variation is given by the black error bars. b. Claim verification accuracy on the evidence retrieval subset of the CIViC-Fact dataset using the BGE passage retrieval model for evidence input. Error bars are given as the range of values (min, max).

It is computationally expensive to train or fine-tune a large language model, therefore recent work has explored ways to improve the output from a language model without fine-tuning or otherwise changing the actual model. Instead, we adjust the input to the model, this is called in-context learning (ICL). One of the most popular ways of doing ICL is by prepending the input prompt with examples of what you would like the model to output and how you would like it formatted. Zero-shot and few-shot^65^ refer to the number of examples that the model is given, 0 and 1 or more respectively. Open LLMs in the zero or few-shot settings performed poorly regardless of size. Few-shot ICL was tested giving the models 1, 3, or 6 examples, but did not result in significant improvements over zero-shot ICL in most cases (data not shown). The highest zero-shot accuracy achieved by an open LLM was Mixtral 70B at 49% (Fig 5a). Proprietary models outperformed all Open LLMs in the zero-shot setting, achieving 72% and 74% for Gemini-2.5 and GPT-4.1 respectively. In contrast, the simpler encoder-based classification models, albeit without reasoning or interpretability capabilities, were only marginally outdone by fine-tuned LLMs in terms of pure classification accuracy. Encoder-based models achieved 86% accuracy, while being far more computationally efficient. Furthermore, both fine-tuned encoder-based models (BERT^59^ and BioMedBERT^23^) outperformed all zero-shot open and proprietary LLMs. This is consistent with previous work demonstrating that despite the gains achieved by LLMs in generating text, encoder-only models remain competitive in text classification^66,67^. Notably, by fine-tuning a smaller open-source LLM (Llama-3.1 8B) on our task-specific dataset, we achieved 89% accuracy, demonstrating that targeted domain adaptation can yield state-of-the-art performance without reliance on proprietary systems.

In practice, the pipeline would be composed of both passage retrieval and claim verification components. Therefore, we also evaluated the classification accuracy when the claim verification model was given evidence from the passage retrieval model (BGE), rather than the evidence manually selected by the expert curators (gold-evidence). Results were similar to the classification accuracy we saw with the gold-evidence and we achieved up to 85% accuracy in stance labelling using the fine-tuned Llama model (Fig 5b).

### Fact-Checking Uncovers Curation Errors in CIViC

In order to demonstrate the utility of our fact-checking pipeline, we fact-checked 1431 entries (including accepted, rejected, and submitted claims) in CIViC where the full-text was available in PMC. CIViC entries were converted to claims and relevant passages were selected from the source publication as evidence using the BGE passage retrieval model. We then grouped the resulting claim/evidence pairs into two categories: those where the LLM consistently produced the correct label during claim verification, and those where the LLM produced at least one incorrect label, which we classified as flagged (Table 3).

We manually reviewed 90 randomly selected claim/evidence pairs to estimate how often CIViC content (both submitted and accepted) requires revision. The overall error rate was 7.8% (7 out of 90). However, errors were far more frequent in entries flagged by our pipeline (28.6%) than in unflagged entries (3.9%), showing that the system reliably highlights problematic content. Extrapolating these estimates to the full CIViC database suggests that more than half of all errors (57.2%) could be identified and corrected by reviewing fewer than one-fifth of entries (15.6%). This targeted review strategy would substantially reduce editor workload while maintaining high data quality.

To further validate this finding, an expert annotator conducted a manual review of the 278 flagged claims. Several of these were excluded either due to the PMC file only containing an abstract and not the full text or due to the claim information not being available in the full text (e.g., requires supplementary tables or image data). Of the remaining 245 entries, 91 (37.1%) were considered actionable, meaning they either were already pending revisions, were rejected, or required revisions. 25 of these flagged entries had already been rejected in CIViC. Of the remaining 66 actionable entries, common errors identified by this analysis included: general human error (24.2%), the wrong level of specificity (43.9%), and variants which should have had a complex molecular profile (MP) (25.8%). General human errors included: the wrong disease being specified (e.g. colorectal cancer instead of melanoma); inclusion of a therapy that was not tested by the source publication; the wrong *Evidence Direction* (e.g. does not support instead of supports); etc. A variant specificity error occurred when “ECSCR expression” was given as the variant instead of “ECSCR overexpression”. Likewise, in another entry, a disease specificity occurred when the disease from a case report was given as “Breast Cancer” instead of the more specific term “Breast Ductal Carcinoma”. Since complex molecular profiles were only added to CIViC in January 2023, it is expected that many older entries now need updates to take advantage of this new feature as more than 85% of the database was entered prior to this.

### LLM Explanations are Promising but Unreliable

**Figure 6.**
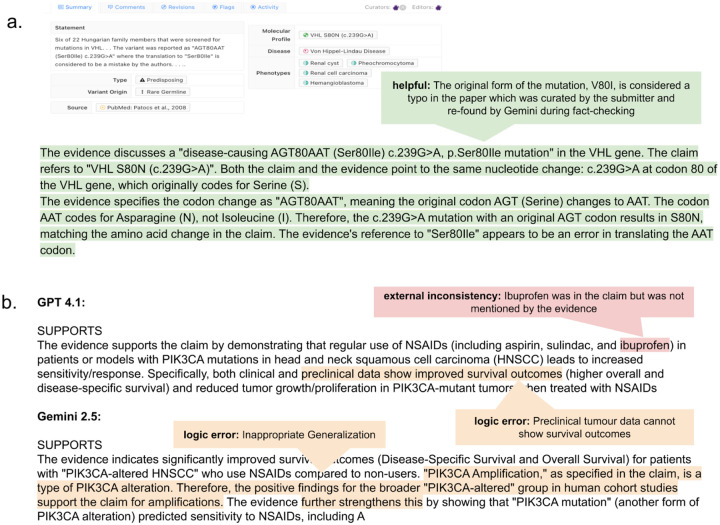
a. Gemini identified a typo in the original paper and used the three-letter codon and CDS notation to verify the variant matched the claim. b. Both Gemini and GPT produced major issues manually annotated by expert human annotators.

Given that the strength of using an LLM for classification lies in the reasoning capabilities, we further evaluated the explanations generated by LLMs. Human experts were asked to tag each LLM response to identify a variety of different errors, or helpful content (Table 1). Some responses were particularly impressive. For example, Gemini-2.5 demonstrated strong logical reasoning by identifying a mistake in the variant name reported in the source publication. The claim listed the variant as S80N, while the publication included S80I, but in reality, all other representations in the source consistently referred to S80N. Gemini detected this discrepancy by translating the three-letter codon into its corresponding amino acid (Fig 6a). At the same time, models also produced coherent but problematic answers. GPT-4.1, for instance, incorrectly claimed that ibuprofen was tested in the source publication; while the claim mentioned ibuprofen, it was not actually included among the therapies studied. Both Gemini and GPT-4.1 also made logically plausible but ultimately incorrect inferences (Fig 6b).

**Figure 7.**
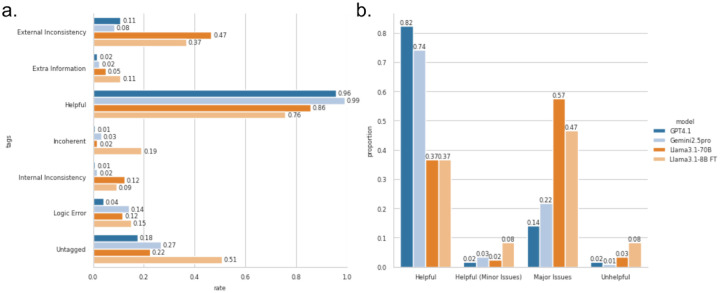
a. Individual rates of tags used by response author (generative model). b. Classification of responses. We categorized responses into four groups based on their tagging results: Major Issues, if any major issues were identified; Helpful (Minor Issues), if the response was considered helpful and only minor issues were tagged; Helpful, if the response was helpful with no issues identified; and Unhelpful for all remaining responses.

The overall inter-annotator agreement among tags was fair (FK: 0.30, PA: 60.4%, p-value: 0.0). When we calculate agreement on the union of the error tags the agreement is considerably higher (FK: 0.44, PA: 74.8%, p-value: 0.0), indicating that while there is some disagreement amongst annotators on the individual type of problem, annotators reach a greater consensus that a problem exists. The lowest agreement was found among untagged content (FK: 0.15, PA: 75,4%, p-value: 4.43×10^−4^) indicating greater disagreement amongst annotators on whether or not content was relevant. Given this level of agreement, each response, along with its initial tags, was re-examined by an additional annotator or collaboratively discussed by the group to resolve disagreements, and to capture additional tags such as “*incomplete”* and “*misleading, or overstated”*. All subsequent results are reported from this re-examined set of annotations. Most models produced responses with some helpful content, but the rates of problematic entries were also quite high (Fig 7a).

GPT-4.1 performed the best in providing helpful responses but still had major issues (factual errors) in 14% of responses. Interestingly, despite being a completely different dataset, this rate of hallucinations is similar to previous work which observed a hallucination rate of 15% on the biomedicine subset of the HaluEval2.0 dataset with ChatGPT^68^. Despite this, proprietary models still considerably outperformed the smaller locally run Llama models. 84% of GPT-4.1 responses were considered helpful with no or only minor issues, compared to 39%-45% of Llama responses (Fig 7b). While fine-tuning resulted in competitive stance classification, it had less of an impact on explanation quality. The larger non-fine-tuned Llama model had 10% more responses with major issues than the fine-tuned smaller Llama model. This analysis is also affected by the small subset of CIViC-Fact with written explanations that limits training of the explanatory component. While we include generated explanations for some NEI claims, these do not substitute for human-written explanations. We continue to collect curated explanations in the CIViC-Fact dataset in hopes to improve LLM reasoning capabilities with further fine-tuning in future versions.

## Discussion

In this work, we introduce a focused fact-checking pipeline to support the verification of cancer variant interpretation claims within the CIViC knowledgebase. Our system targets a critical need in precision oncology: verifying literature-backed assertions with NLP tools. By concentrating on passage ranking and claim verification, our approach directly supports curators reviewing claims whose provenance, i.e., the associated document, is already known, as is the case in the manually-curated and literature-derived CIViC. Our contributions span dataset creation, model development, system evaluation, and human-centered analysis, laying the groundwork for scalable and reliable automated fact-checking in precision oncology. This dataset introduces new opportunities for automated systems to be trained and evaluated in a way that closely mirrors expert human workflows.

A central contribution of our work is the creation of a novel benchmarking dataset that links structured claims in a precision oncology knowledgebase (CIViC) to sentence-level evidence provenance from the primary literature. This dataset enables the rigorous evaluation of fact-checking models and introduces a new resource for the community focused on verifiable, high-stakes biomedical claims. Building on this dataset, we implemented a focused end-to-end fact-checking pipeline that includes passage ranking, and automated claim verification. We experimented with a range of passage ranking models and trained large language models (LLMs) specifically for biomedical claim verification. Although resource constraints limited our testing of the largest open LLMs, fine-tuning a relatively small LLM (Llama3.1 8B) paired with the BGE retrieval model is able to outperform larger and proprietary models at fact-checking by a wide margin (85% vs 59%).

Beyond model evaluation, we used the CIViC-Fact pipeline to flag potentially incorrect entries in the CIViC knowledgebase itself. By applying the fact-checking pipeline and manually reviewing cases where predictions conflicted with curated claims, we identified several instances of curator error, or errors resulting from schema changes to the database since an entry was created. These flagged entries were returned to the CIViC curation team for follow-up, resulting in corrections to knowledgebase content. While human error accounted for nearly a quarter of these errors, many were also found in previously reviewed content and were attributable to schema changes, such as newly introduced support for complex molecular profiles, that occurred after the initial curation. Allowing Schema changes is essential for improving knowledgebase content but, as we have demonstrated, this can result in even previously accepted and reviewed content requiring updates that may not be easily automatable. Having a fact-checking system that can flag such entries improves efficiency and keeps older knowledgebase content to a high level of accuracy. These findings demonstrate the practical utility of CIViC-Fact and underscore the importance of continuous knowledgebase auditing as data models evolve and user needs shift, further highlighting the need for automated curation support.

Crucially, we performed a fine-grained review of not only the LLM-generated labels, but also their associated explanations. Our findings indicate that while most of the LLMs tested produce some helpful rationales, their factual consistency and alignment with domain expectations leave room for improvement. In particular we see the impact of model size here with the larger proprietary LLMs significantly outperforming smaller open LLMs. This highlights both the promise and current limitations of LLM-based explanations in sensitive domains such as clinical genomics.

Overall, our work demonstrates the feasibility and utility of integrating NLP-based fact-checking tools into the CIViC curation pipeline. This augmentation has the potential to reduce curator burden, accelerate literature review, and flag questionable claims for further human inspection. However, our results also underscore the need for ongoing evaluation of model outputs, especially in contexts where interpretability and accountability are critical.

## Limitations and Future Work

While our system shows promising results, several limitations remain. First, we only locally tested smaller quantized models, while this is reasonable given that likely any centre would need to account for resource constraints it is not a strictly fair comparison of the open LLM models capabilities. We also only consider evidence in the main text of the article, while this is an improvement over limiting to the abstract, future iterations should leverage images and supplementary materials. We are currently collecting instances of CIViC entries that require images or supplementary material but they are too small a proportion of the dataset yet to properly evaluate a model on them (<10%).

We envision future extensions of this work that integrate additional annotator feedback, incorporate uncertainty quantification in model predictions, and expand the scope of verifiable claims to include multi-modal evidence and supplementary materials. With continued development, we believe this approach can help foster more trustworthy, evidence-backed cancer knowledgebases.

## Conclusions

Our findings raise important questions about the role of language models in biomedical information pipelines. While open LLMs offer exciting possibilities for automation, they are not yet reliable, particularly when applied to high-stakes domains like precision oncology. Their low accuracy in the absence of fine-tuning or high resource requirements, inability to consistently justify their answers without error, combined with hallucinations and biomedical knowledge gaps, limits their utility for trust-critical applications. We highlight how human-AI collaboration can help verify scientific knowledge by using AI fact-checking pipelines as first-pass filters, prioritizing questionable claims for expert review. Instead of replacing human curators, these systems can amplify expert effort, improving curation throughput and quality. Resources like CIViC-Fact offer a way forward by creating evaluation datasets based on real biomedical claims with traceable evidence, helping us build models that are both accurate and accountable.

## Figures and Tables

**Table 1.** Definitions of tag categories and types used in evaluating LLM response text.

Category	Type	Definition
Helpful		Text is directly relevant to the claim/evidence pair in question and is useful and specific in explaining why/how the stance of the evidence is SUPPORTS, REFUTES, or NEI toward the claim. Should go beyond simply restating the claim.
Major Issues	external inconsistency	Text contradicts facts of the claim or evidence.
	internal inconsistency	Text contradicts itself.
	logic error	Text makes an illogical inference or assumption.
Minor Issues	extra information	Text includes facts, citations, or numbers that cannot be derived from the claim or evidence. General biomedical knowledge is acceptable only if it does not require citation
	incoherent, poor wording, or poor grammar	Text is difficult or impossible to understand due to confusing wording or structure. OR The text is understandable but poorly worded or ungrammatical in a way that makes it sound unnatural or unlike human writing.
	Incomplete	The response omits important details that would be expected in a strong human-written explanation.
	Misleading or Overstated	Text is technically plausible but could be easily misinterpreted, or it uses inappropriate qualifiers that make the conclusion stronger or weaker than warranted.
Unhelpful	Untagged	Text left unselected by curators is assumed to be irrelevant, repetitive, non-specific or otherwise not helpful

**Table 2.** Quantitative characteristics in each split of CIViC-Fact Claim Verification dataset

		train	dev	test
Gold Label	SUPPORTS	2162	712	689
	REFUTES	2091	676	666
	NEI	2175	711	700
Claim	CIViC	2854	961	943
	Modified	2236	764	679
	Generated	1338	374	433
Evidence	Full-text	1941	662	557
	Abstract	1192	346	399
	CIViC Statement	3295	1091	1099
Validation Review	yes	170	96	83
	no	6258	2003	1972
***Total***		6428	2099	2055

**Table 3.** CIViC claims evaluated via the CIViC-Fact fact-checking pipeline.

			Total		Errors		Error Details (n=66)		
Dataset	total	Flagged	n	%	n	%	type	n	%
Random Selection	90	yes	14	15.6	4	28.6	--	--	--
		no	76	84.4	3	3.9	--	--	--
CIViC (Full-text)	1431	yes	278	19.4	91	37.1% (of 245)	complex MP	17	25.8
							specificity	29	43.9
							human error	16	24.2
		no	1153	80.6	--	--	--	--	--

## Data Availability

Language Models Versions of the CIViC-Fact dataset can be downloaded from github (https://github.com/creisle/civicfact) along with unprocessed data from manual reviews. A frozen download of the CIViC *Evidence Items* used in this analysis is also included for reproducibility. Code for building the dataset and running language model experiments can be found on github (https://github.com/creisle/civicfact) as can the forked version of doccano^44^ with additional feature support used for the manual review tasks (https://github.com/creisle/doccano).
